# Precision Medicine for Pediatric Glioma and NF1-Associated Tumors: The Role of Small Molecule Inhibitors

**DOI:** 10.3390/curroncol32050280

**Published:** 2025-05-15

**Authors:** Samuele Renzi, Julie Bennett, Nirav Thacker, Chantel Cacciotti

**Affiliations:** 1 Division of Pediatric Hematology/Oncology, CHU de Québec-Université Laval, Québec City, QC G1V 0E8, Canada; samuele.renzi.med@ssss.gouv.qc.ca; 2 Department of Pediatrics, CHU de Québec-Université Laval, Québec City, QC G1V 0E8, Canada; 3 Division of Hematology/Oncology, The Hospital for Sick Children, Toronto, ON M5G 1E8, Canada; julie.bennett@sickkids.ca; 4 Arthur and Sonia Labatt Brain Tumour Research Center, Toronto, ON M5G 0A4, Canada; 5 Division of Medical Oncology and Hematology, Princess Margaret Cancer Centre, Toronto, ON M5G 2C4, Canada; 6 Division of Hematology/Oncology, Children’s Hospital of Eastern Ontario (CHEO), Ottawa, ON K1H 8M8, Canada; nthacker@cheo.on.ca; 7 Division of Hematology/Oncology, Department of Pediatrics, London Health Sciences Centre & Western University, London, ON N6A 5W9, Canada

**Keywords:** glioma, MEK inhibitor, BRAF inhibitor, Pan-RAF inhibitor, FGFR inhibitor, TRK inhibitor, NF-1, plexiform neurofibroma, tuberous sclerosis, mTOR inhibition

## Abstract

Pediatric gliomas encompass the most common brain tumor in children and are subdivided into pediatric low-grade gliomas (pLGGs) and pediatric high-grade gliomas (pHGGs). The era of molecular diagnosis has shifted the treatment paradigms and management of these patients. RAS/MAPK pathway alterations serve as the driver in the majority of pLGGs, a subset of pHGG and NF1-related plexiform neurofibromas (PNs). The role of small molecule inhibitors in the treatment of these tumors has evolved in the past decade, facilitated through multiple clinical trials and moving into earlier stages of treatment. Although these developments hold promise, questions remain regarding targeted therapy, the long-term toxicities, the duration of treatment and the potential effects on the natural history of the tumor behavior.

## 1. Introduction

Central nervous system (CNS) tumors are the second most common type of cancer and the leading cause of cancer-related death in children. Of these, gliomas are the most common type of tumor found in the pediatric age group. Glioma is subdivided into pediatric-type low-grade glioma (pLGG) and pediatric-type high-grade glioma (pHGG), with this distinction made based on morphology and in some cases by specific molecular alterations in the tumor as described in the 2021 WHO classification of tumors of CNS [1]. Gliomas can be found throughout the CNS. These tumors extend beyond pediatrics and can be seen in adults.

Over the past 15 years, many breakthroughs have been made to understand the molecular drivers of human cancers. This has led to new therapeutic options, including targeted therapy. This involves targeting specific cellular pathways to reduce cellular proliferation, either at the level of aberrant protein production or downstream. An example is neurofibromatosis type-1 (*NF1*), where patients have a germline alteration in NF1, and tumors are thought to form with a second somatic mutation leading to inactivation of the NF1 protein. This leads to constitutive activation of RAS/RAF/MEK/ERK, thereby opening up the possibility of using small molecule inhibitors to target this pathway. In this review, we will describe the current landscape of targeted therapy, specifically RAS/MAPK inhibitors, in the treatment of pediatric gliomas and plexiform neurofibromas.

## 2. Low-Grade Glioma

Pediatric low-grade gliomas (pLGGs) represent the most common CNS tumor in children [2]. These tumors are heterogenous and encompass various histological subtypes with pilocytic astrocytoma being the most common. Treatment options have evolved for pLGGs. Historically, the mainstay of treatment included surgical resection and adjuvant radiation. Since 1980, the role of chemotherapy was introduced with the aim to delay or omit radiation therapy particularly in younger children with unresectable tumors [3,4]. The recent introduction of targeted therapies has further expanded the treatment options.

**Figure 1:**
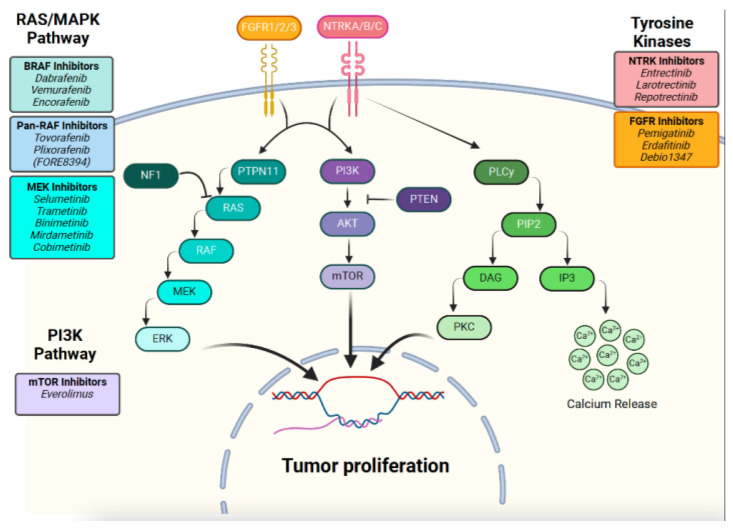
RAS/MAPK and PI3K pathways with relevant targeted therapy agents.

Despite an excellent long-term survival, tumors can be associated with morbidity, with a correlation with tumor location. Treatment includes maximal-safe surgical resection. For those with residual tumor and in need of further treatment, especially those arising in the optic pathway, or midline locations, systemic therapy can be offered. Historically unresectable tumors are treated with adjuvant chemotherapy and, in rare circumstances, radiation. Progression-free survival (PFS) is ~50%, regardless of chemotherapy regimen, with up to half of patients requiring additional therapy; thus, pLGGs can be a chronic condition with multiple relapses/recurrences over time. More recently, the molecular underpinnings of pLGGs have provided additional treatment strategies. Upregulation of the MAP kinase (MAPK) pathway has been identified as a driver of the vast majority of pLGGs, typically as a single alteration driving gliomagenesis. As a result of this knowledge, alongside the development of small molecule inhibitors targeting the RAS/MAPK pathway (Figure 1), the role of small molecule inhibitors in the treatment of pLGGs has been extensively studied [5,6] (Table 1). Sporadic pLGGs can harbor fusions and single nucleotide variants (SNVs) of the oncogene *BRAF*, with *KIAA1549::BRAF* being the most frequent alteration, followed by BRAF p.V600E in a smaller subset. Other alterations in pLGGs include somatic alternations in *FGFR1/2/3*. Furthermore, mutations in the RAS/MAPK pathway suppressor *NF1* can be found in patients with germline alterations (NF1) or rarely as a somatic alteration. pLGGs have distinct biology from their adult counterparts, with adult-type diffuse gliomas containing presence or absence of SNVs in *IDH1/2*, for instance. Furthermore, some pLGGs contain specific drivers that are not amenable to targeted treatments, such as *MYB/MYBL1* alteration) or drivers that may be targetable but more common in adult populations, such as *IDH1/2*.

### 2.1. MEK Inhibitors

MEK inhibition has emerged as a promising therapeutic strategy. MEK inhibitors (MEKis) can be used for tumors harboring *BRAF* fusions or SNVs. Various MEKis are considered, including selumetinib, trametinib, binimetinib, cobimetinib and mirdametinib.

A Pediatric Brain Tumor Consortium (PBTC) phase 1/2 study evaluated the use of selumetinib (PBTC-029) [28]. This initial study established the recommended phase 2 dose of selumetinib (25 mg/m2/dose twice daily). This led to a phase 2 study of selumetinib, a selective MEK1/2i in relapsed and refractory pLGGs, which remains one of the largest published MEKi studies in pLGGs and demonstrated a 30–40% image response rate in recurrent and progressive pLGGs [8]. This included patients with non-NF1 pilocytic astrocytoma with *KIAA1549::BRAF* or BRAF p.V600E mutation (stratum 1), NF1-associated pLGGs (stratum 3) and those with recurrent/progressive non-NF1 optic pathway and hypothalamic pLGGs (stratum 4) [7,8].

Stratum 1 included 25 patients, of whom 18 had *BRAF* fusion and 7 BRAF p.V600E. A central review demonstrated complete or partial response (CR/PR) in eight patients (32%), with a median time to PR of 7.54 months. Ten patients (40%) had stable disease (SD), and seven (28%) developed progression. The two-year PFS was 70% with outcomes worse in BRAF p.V600E vs. *BRAF*-fused patients (~60% vs. 80%, respectively) although not statistically significant. An additional 25 patients enrolled on stratum 3 (NF1 patients). CR or PR was seen in nine patients (36%) with a median time to response of 3.57 months. The 2-year PFS was 96%. In stratum 4 (non-NF-1 optic pathway glioma), 25 patients were enrolled. Six (24%) had PR and fourteen (56%) had SD, with 5 (20%) having progression while on therapy. The 2-year PFS was 78%. Functional outcomes were reported with improvement in visual acuity in 21% (4/19) and improvement in visual fields in 26% (5/19). Notably on this stratum, biopsy was not required. Only six tumors underwent genetic testing, with *BRAF* fusion identified in three. Out of the 75 patients, toxicity resulted in dose reduction for 29 patients and discontinuation of therapy for nine children [7].

Until recently, selumetinib was suggested to be administered twice daily (BID) under fasting conditions, 2 h fasting before and 1 h fasting after, which is inconvenient and results in up to 6 h of fasting per day. More recently, studies have demonstrated that selumetinib dosing with a low-fat meal had no clinically relevant impact on AUC, thus suggesting that fasting was not required [29].

Trametinib is an oral MEK1/2 inhibitor with once daily dosing that is approved for the treatment of patients with melanoma, non-small cell lung cancer and anaplastic thyroid cancer [30,31,32,33,34]. Trametinib is a reversible, highly selective inhibitor of MEK1/MEK2 activation. It is recommended that trametinib be taken under fasting conditions, 1 h before or 2 h after a meal given that food can reduce the AUC by 24% and Cmax by 70% [11]. Trametinib demonstrated a 15% response rate in patients with recurrent BRAF p.V600E tumors in a phase 1 prospective trial. The combination of trametinib and dabrafenib in BRAF p.V600E pLGGs demonstrated a 25% response rate with no dose limiting toxicities. There are ongoing studies evaluating trametinib use [9]. TRAM-01 is a phase II study evaluating trametinib in progressive/refractory gliomas with MAPK/ERK pathway activation (NCT03363217) [11].

Fewer studies have evaluated binimetinib and cobimetinib. Binimetinib is an oral, selective, MEK1/2 inhibitor approved for the treatment of patients (adult and pediatric) with unresectable or metastatic melanoma [35]. Binimetinib (MEK162) was evaluated in a phase II study of progressive or recurrent pLGGs and demonstrated a 56% radiographic response. Although the results of this trial are not finalized, the rates of dose reduction or discontinuation seemed to be higher with binimetinib compared to other MEKis (49% and 22%, respectively). This study has suggested that binimetinib may be effective in NF1-associated and sporadic pLGG patients (with or without BRAF fusions) [12].

A phase I/II study (iMATRIX-cobi) evaluated cobimetinib in pediatric and young adult patients with relapsed or refractory solid tumors) [13]. This included 56 patients, 32 of whom had LGGs. In LGG patients with reported MAPK pathway alterations, the ORR was 13% (3/23). This is less than what has been reported with other MEK inhibitors.

Mirdametinib, an oral small molecule MEK 1/2 inhibitor approved for the treatment of plexiform neurofibroma in pediatric and adult patients has also more recently been used in children, adolescents and young adults with LGGs. Preliminary results from the ongoing Phase 1/2 trial (NCT04923126) suggest that mirdametinib may be beneficial in patients with recurrent or progressive LGGs [36]. This study included 23 patients, 74% (17/23) completed or remained on therapy, 17% (4/23) discontinued due to progression and 2% discontinued due to toxicity [36]. Median time to objective response was 5.4 months, with 63% (12/19) patients with measurable tumors achieving an objective response [36].

The concept of combination therapy is an ongoing area of study in pLGGs. It remains unclear whether unresectable pLGGs would benefit from a combination approach or single-agent targeted therapy. There are ongoing studies posing these questions. PNOC201 is evaluating the combination of an MEKi (trametinib) and mTORi (everolimus), whereas the Children’s Oncology group (NCT04166409/NCT03871257 or ACNS1831/1833) is currently evaluating the role of MEKis in newly diagnosed and previously untreated optic pathway/hypothalamic gliomas either as a single agent or in combination with chemotherapy, standard chemotherapy, carboplatin and vincristine.

### 2.2. BRAF Inhibitors

BRAF inhibitors (BRAFis) include dabrafenib, vemurafenib and encorafenib. These agents have shown excellent results in melanoma patients with BRAF p.V600E and more recently in pLGGs. BRAF p.V600E has been found in approximately 20% of pLGGs, enriched within ganglioglioma and pleomorphic xanthoastrocytoma. BRAFis are contraindicated in those with NF1-associated LGG and *BRAF* fusions due to paradoxical pathway activation.

Dabrafenib is a BRAFi that has been shown to induce favorable radiographic responses in relapsed/refractory pLGGs with BRAF p.V600E in phase 1/2 safety and efficacy trials [37]. Radiographic response was rapid with a median time to first response of 3.8 months and with a median duration of response of 26 months. Efficacy analysis in these trials included 32 pLGG patients with BRAF p.V600E, demonstrating an overall response rate (ORR) of 44%, with 1 patient having a complete response (CR) and 13 partial responses (PR) per RANO criteria [14]. Treatment was overall well tolerated [14]. A phase I prospective trial demonstrated a 15% response rate for trametinib alone and 25% for combination therapy in recurrent BRAF p.V600E pLGGs [9]. These promising results led to the initiation of upfront targeted therapy trials. In a phase II trial comparing dabrafenib plus trametinib to standard chemotherapy as an upfront treatment for BRAF p.V600E pLGGs, therapy showed clear benefits: higher response rates (47% vs. 11%, respectively), longer PFS (20.1 vs. 7.4 months, respectively) and better safety profile (grade 3 or higher AE 47% vs. 94%, respectively). These findings support the use of upfront targeted therapy in pLGGs with BRAF p.V600E mutations [10].

Similarly, vemurafenib has demonstrated favorable and rapid responses with a time to best radiographic response of 3 months and mean sustained response time 2 years [16]. In a phase I study of recurrent/refractory *BRAF* p.V600E mutant pLGGs, vemurafenib demonstrated a CR in 1 patient and PR in 5, with the remaining 13 showing stable disease (SD) on central radiology review [15]. Another retrospective study showed a 57% response rate to single-agent vemurafenib, when utilized mainly as an initial therapy (6/7) [16]. Within this group, responses included CR in 1, PR in 2 and SD in 1.

Larger international retrospective cohort studies have shown 80% objective radiographic responses in pLGGs with a median time to best response of 4 months [17]. This included 67 patients with BRAF V600E-mutated gliomas, 56 pLGGs and 11 pHGGs [17]. Patients were treated with either dabrafenib or vemurafenib. Notably, 13 out of 17 pLGG patients experienced rapid progression (median 2.3 months) after discontinuing BRAF inhibition. However, upon re-treatment with BRAF inhibitors, 90% achieved an objective response.

Unanswered questions remain in the context of MAPKi therapy. The duration of therapy required remains elusive. The cessation of these agents poses the risk of rapid recurrence, although a re-introduction of therapy tends to demonstrate response. Studies are needed to identify the appropriate duration of therapy and how to safely discontinue treatment to minimize the neurologic consequences of tumor progression. There have been consensus recommendations suggesting a slow tapering approach when discontinuation of therapy is being considered to avoid the associated rapid clinical and radiographic progression [38]. In addition, the International Pediatric Low-Grade Glioma Coalition has formed a resistance, rebound and recurrence working group aimed at creating consensus-based guidelines to address these specific concerns when treating pLGGs with targeted therapy [39].

### 2.3. Next-Generation RAF Inhibitors

Tovorafenib (also known as DAY101, TAK-580 and MLN 2480) and Plixorafenib (FORE8394) are two agents that also inhibit the RAS-MAPK pathway. Tovorafenib is an oral CNS penetrant, small-molecule pan-RAF kinase type II inhibitor that has demonstrated success in the treatment of pLGGs [18], whereas Pilxorafenib (FORE8394) is a newer agent that inhibits BRAF p.V600E and non-V600 alterations, while avoiding paradoxical MAPK activation.

Plixorafenib is currently under investigation in clinical trials in patients with BRAF-altered solid tumors. A phase 1/2 study’s (NCT02429712) preliminary results have demonstrated a 42% overall response rate and 17.8-month median duration of response with Plixorafenib in MAPKi-naive patients with BRAF p.V600E-mutated solid tumors [20]. Amongst these were nine adult patients with CNS tumors, ORR 67% and mean duration of response 13.9 months. Plixorafenib is also being investigated in an ongoing phase 2 study in children and adults with BRAF alterations, including LGGs and HGGs (FORE study, NCT05503797).

FIREFLY-1 (PNOC026), a phase 2 trial demonstrated an 83% clinical benefit rate (a stable disease of any length of time) with tovorafenib, in patients with BRAF-altered (*n* = 77; arm 1) and RAF-altered (*n* = 60; arm 2), relapsed or refractory pLGGs. Participants had a 51% overall response rate based on RAPNO, a median duration of response 13.8 months and a median time to response of 5.3 months. Treatment was overall well tolerated. Furthermore, the clinical benefit was noted in *BRAF*-altered relapsed/refractory pLGGs with tovorafenib in more than half of the patients previously treated with RAF and/or MEKis, suggesting a valid option in those who failed prior targeted therapy.

Ongoing clinical trials utilizing tovorafenib are underway, including a phase II trial (NCT05566795) of tovorafenib monotherapy compared to standard chemotherapy (investigators’ choice based on the standard of care—Vincristine/Carboplatin or Vinblastine) in upfront pLGG treatment [19]. In addition, a multicenter VICTORY trial (NCT06381570) is evaluating the use of tovorafenib in combination with vinblastine chemotherapy in recurrent/progressive RAF-altered pLGGs.

### 2.4. FGFR Inhibitors

Erdafitinib is a pan-FGFR inhibitor that initially showed significant efficacy in adult urinary tract malignancies harboring FGFR2/3 mutations. Its utilization in pLGGs, characterized by FGFR1 mutations, has been increasing. Preliminary results of the COG MATCH trial (NCT03210714) have shown a promising efficacy of this drug in pLGG patients whose tumor harbors a FGFR1 rearrangement as 54% of the patients had either partial response or stable disease [25]. However, the recent results from the phase 2 RAGNAR trial, which included patients aged 12 years or older, did not demonstrate any investigator-assessed objective response in the low-grade glioma cohort, even if one should consider the very limited number of patients included in this study, most of whom had progressive/refractory tumors [40].

Pemigatinib is a selective oral FGFR1-3 inhibitor that seems to have better blood–brain barrier penetrance when compared to erdafinitib. Large studies investigating the efficacy of this drug in the pediatric population are lacking, hence data are often extrapolated from adult trials. The FIGHT-207 phase 2 trial involving adult CNS patients with both low- and high-grade gliomas showed partial or complete response in 23% of the patients, with another 23% having stable disease while on treatment. The subsequent ongoing phase II trial FIGHT-209 is currently investigating the activity of pemigatinib in adult patients with recurrent FGFR1-3-rearranged solid or CNS tumors (NCT05267106) [41].

### 2.5. TRK Inhibitors

Genetic rearrangements involving genes of the NTRK family are known to be present in various adult and pediatric CNS and solid tumors; while their occurrence remains extremely rare in adult patients as it is estimated to be less than 2%, they can be found in over 5% of pediatric patients with high-grade glioma and in up to 3% of pediatric patients with low-grade glioma [23,42]. A recent international retrospective study including pediatric and adult patients with TRK fusion-driven CNS tumors showed that half of the patients were infants. Furthermore, the outcomes for the pediatric patients are significantly better when compared to adults; not surprisingly, LGGs have improved outcomes compared to HGGs [23].

Larotrectinib is a selective tropomyosin receptor kinase that has been approved for the treatment of TRK-driven malignancies, including TRK-altered primary CNS tumors. A large multicenter, open-label, phase 1 study showed that the drug had an overall good toxicity profile, with a significant antitumoral activity, as 93% of the patients had an objective responses as per the Response Evaluation Criteria in Solid Tumors version 1.1 [43]. However, when narrowing it down to the CNS cohort, the results are clearly inferior. In a recent pediatric and adult series including 55 patients with a primary CNS tumor TRK-rearranged, ORR for pediatric LGGs was 42%. Of note, the median duration of response (DoR) for pediatric patients was 17 months, with a 3-year DoR rate of 37% [27]. The efficacy of this drug, particularly in infants with high-grade glioma and a NTRK-rearrangement, has been confirmed by several studies [23,27].

Entrectinib is a tyrosine kinase inhibitor that targets TRK-A, TRK-B, TRK-C, ROS1, and ALK. A recent international phase 1-2 study (STARTRK-NG) confirmed its efficacity in pediatric and young adult patients with a TRK-rearranged solid or CNS tumors, with an overall response rate in the CNS cohort of 50% [24]. Several reports have showed its efficacity and safety in pediatric CNS and solid tumors harboring an NTRK fusion [44,45].

Repotrectinib is a next-generation ROS1 inhibitor with good brain penetrance; it has been recently FDA approved for adult and pediatric patients aged 12 years of older whose solid and or CNS tumor harbor a NTRK rearrangement; this was mainly due to the results of the TRIDENT-1 phase 1/2 adult clinical trial with patients whose tumors were characterized by a NTRK gene fusion, with an objective tumor response in half of the cohort [46]. A phase 1/2 open-label clinical trial is currently investigating the efficacy of repotrectinib in children and young adults diagnosed with an advanced or metastatic tumor harboring ALK, ROS1, or NTRK-1-2-3 alterations (NCT04094610).

### 2.6. m-TOR Inhibition

Inhibition of the mammalian target of rapamycin (mTOR) signaling pathway remains another therapeutic target for CNS tumors. pLGGs demonstrate an abnormal signaling upstream of mTOR through mutations in receptor tyrosine kinases or alterations in BRAF [22,47]. The POETIC phase 1-2 study suggested that everolimus could grant some disease control in recurrent and or progressive pediatric low-grade gliomas [22,48].

The results from the phase II PNOC001 trial confirmed that everolimus can be an option for this same indication. Furthermore, a phase 2 study involving 23 pediatric and young adult patients with recurrent, radiographic progressive NF1-associated pediatric low-grade glioma showed that continuous oral everolimus was effective in stopping tumor growth and or obtaining tumor reduction in 68% of the patients [49]. The ongoing phase 1 PNOC021 study is investigating the role of combining everolimus and trametinib in pediatric and adult patients with a recurrent low-grade gliomas (NCT04485559) [22].

## 3. Tuberous Sclerosis

Patients with tuberous sclerosis have an inherited hyperactivation of the mammalian target or rapamycin (MTOR) pathway as the two genes mutated in this disease; TSC 1 and TSC2 are an onco-suppressor that act as a negative regulator of this pathway. As a result, besides the other clinical manifestations of this syndrome, patients have a higher risk of developing subependymal giant-cell astrocytoma (SEGA), which are low-grade astrocytic tumors that generally occur in the ventricles and are often associated with the occurrence of seizures [50].

### m-TOR Inhibition

Everolimus is a m-TOR inhibitor that has become the standard of care treatment for the treatment of SEGA tumors as a result of an open-label study on patients 3 years of age and older (NCT00411619). This has shown a significant reduction in both the volume of the SEGA and the frequency of seizures [51]. A subsequent >5 years analysis derived from this same study confirmed these same findings, with a sustained therapeutic response in over 50% of patients, along with a good safety profile of the drug [52].

## 4. High-Grade Glioma

High-grade gliomas are associated with poor prognosis despite surgical advances and aggressive treatment with chemo-radiotherapy. Treatment typically involves maximal safe resection followed by focal radiation with concurrent chemotherapy (TMZ), followed by adjuvant chemo, typically TMZ-based regimens with the possible addiction of lomustine [53,54].

pHGGs are distinct molecularly from their adult counterparts and recognized as a discrete subgroup within the 2021 WHO CNS tumor classification. In children, pHGGs tend to more frequently encompass mutations in PDGFRA, TP53 and recurrent K27M and G34R/V mutations (Table 2). Approximately 5 to 10% of pHGGs are driven by somatic MAPK pathway alterations, most commonly point mutations in BRAF oncogene [55,56,57].

### 4.1. MEK Inhibitors

Single-agent MEKi use has not shown as promising results in pHGGs; this is postulated to be related to multiple gene alterations and drivers in pHGGs in comparison to a single-gene alteration in pLGGs resulting in more encouraging results with MEKis. The Pediatric MATCH trial evaluated a MEKi (selumetinib) in patients with MAPK pathway alternation, including seven pHGG patients [58]. Stable disease was noted in two pHGG patients, although no patients demonstrated objective response. Selumetinib treatment in pHGG and rhabdomyosarcoma patients was associated with a 15% 6-month PFS in this study [58].

### 4.2. BRAF Inhibitors

Single-agent BRAFis or BRAFis in combination with MEKis have been shown to have more favorable responses in the management of recurrent and upfront pHGGs. Although in contrast to children with LGGs, targeted therapy in recurrent BRAF-mutated pHGG patients remains less efficacious with a reported median PFS of approximately 3 months [17].

In refractory, recurrent or progressive BRAF V600E-mutated pHGGs, a phase 1/2 study (NCT01677741) demonstrated an ORR was 45% with median duration of response of 7.7 months, which remains superior to other published reports. Amongst these patients, 13/19 (68%) demonstrated a 50% or greater reduction in tumor [14]. In contrast, combination therapy (dabrafenib and trametinib) treatment in relapsed refractory pHGGs demonstrated an overall better response rate (56% vs. 54%) when compared to single-agent dabrafenib. Combination dabrafenib plus trametinib in a phase 2 study of relapsed/refractory BRAF V600-mutated pHGGs included 41 patients, with a median follow up of 25.1 months, at which time 51% remained on therapy [60]. ORR was 56% by RANO criteria, with a complete response in 29% (12 patients) and partial response in 27% (11 patients). Most responded within 4 months of therapy, with approximately 90% having a 50% reduction in tumor size. Progressive disease was noted in 16/20 patients necessitating a discontinuation of therapy. This study demonstrated encouraging PFS and OS results with a median PFS and OS of 9 and 32.8 months, respectively, and suggests a promising treatment option for relapsed/refractory pHGGs with BRAF V600 mutations.

Other studies have also shown encouraging results with the use of BRAF +/− MEK inhibitors as upfront therapy. An international retrospective analysis included 8 patients treated with BRAFi monotherapy and an additional 11 treated with BRAFis and MEKis. Of those with measurable disease, 13 patients, eight responses were noted (two with CR and six with PR) with a median time to best response of 2.5 months and sustained response >6 months [61]. In comparison to historical chemotherapy treated controls, improvement was noted with a 3-year PFS and OS was 65% and 82%.

Given these encouraging results of upfront treatment in pHGGs with BRAF p.V600E, additional studies are underway. One such trial (NCT03919071) is investigating dabrafenib and trametinib following radiation therapy in newly diagnosed pHGG patients. Another study (NCT04201457) is exploring the use of hydroxychloroquine in combination with trametinib (for BRAF fusion or NF-1 associated glioma) or with trametinib and dabrafenib (for BRAFp.V600E) in recurrent pLGGs or pHGGs). Although the combination of hydroxychloroquine with dabrafenib/trametinib has shown limited success in melanoma [62,63], data in glioma patients remain to be determined [64].

### 4.3. m-TOR Inhibition

More recently, everolimus has been utilized off-label to treat pHGGs; the BIOMEDE 2.0 study (NCT05476939) is currently investigating the utilization of everolimus vs. dordaviprone (ONC 201) in patients with infiltrating diffuse midline gliomas (DMGs) and diffuse intrinsic pontine gliomas (DIPGs). Everolimus has also been combined with CDK4/6 inhibitors (ribociclib) for the same indications, and the treatment of DIPG and DMG patients, and has demonstrated a good safety profile.

### 4.4. CDK4/6 Inhibition

Abnormal activation of cyclin-dependent kinases (CDKs) is frequently found in neoplastic cells and plays a role in tumorigenesis [65,66]. CDK4/6 inhibitors (abemaciclib, palbociclib and ribociclib) are oral molecules that have shown encouraging results in the treatment of hormone positive advanced breast cancer [67,68,69,70]. In addition, trials are investigating the use in brain metastasis [67,69], meningiomas [71] and other CNS tumors [72,73,74] given good blood–brain barrier penetrance [70,75]. The evidence remains limited for CNS lesions and warrants further research [76]. From a safety perspective, the main side effects reported are hematological (neutropenia and lymphopenia) and non-hematological, specifically diarrhea and hepatobiliary toxicity [77]. Furthermore, there is a reported risk of cardiac adverse events, including atrial fibrillation and acute myocardial infarction [78].

Unanswered questions remain in the context of targeted therapy. The duration of treatment required remains unknown, and the cessation of these agents has led to rapid tumor growth, although response is typically noted on re-initiating of medication; thus, further studies are necessary.

## 5. Neurofibromatosis Type 1 and Plexiform Neurofibromas

NF1 is an autosomal dominant tumor predisposition syndrome with a prevalence of approximately 1 in 3000 [79]. Plexiform neurofibromas (PNs) are benign peripheral nerve tumors that occur in up to half of patients with NF1 and can cause significant morbidity [80]. Depending on the location, size, and rapidity of growth, NF1-PNs can lead to significant pain, disfigurement, motor dysfunction, airway obstruction, visual impairment and bladder/bowel dysfunction [81], thereby needing treatment. In addition, 8–13% of PNs can transform to malignant peripheral nerve sheath tumors, which is one of the leading causes of death in people with NF1 [79].

Until recently, surgical resection was the most effective treatment option and remains the only curative option for symptomatic PNs. Complete surgical excision of PNs is often challenging and can be associated with significant morbidity due to the proximity with vital structures, diffuse nature and profuse vascularity making more than 50% of PNs inoperable [82].

Understanding of the underlying molecular landscape, i.e., the constitutive activation of the RAS/RAF/MEK/ERK pathway secondary to NF1 gene alterations and the availability of small molecules targeting the pathway, provided the perfect opportunity for the beginning of the era of targeted therapies in NF1-PNs, although at this present time, only two drugs are approved by the FDA, selumetinib and mirdametinib (Table 3).

### 5.1. MEK Inhibitors

While multiple MEK inhibitors and tyrosine kinase inhibitors have been investigated in clinical trials in children and adults with NF1-PNs, MEK inhibitors have shown the most promise.

Selumetinib is the first (2020 US-FDA) and the only MEK1/2 inhibitor till date that has been approved for NF1-PNs. Selumetinib has been studied most extensively with four seminal papers from a combined phase I/II SPRINT trials in children aged 2–18 years with inoperable PNs [85,86,87,88]. The SPRINT trial was comprised of two strata: stratum 1 for symptomatic PNs with at least one PN-related complication and stratum 2 for PNs at risk of developing morbidity. The primary endpoint of the study was ORR, which was measured by volumetric MRI, with a ≥ 20% decrease in PN volume defined as partial response (PR) in this trial. Across phase I and phase II (stratum 1), a total of 74 patients were enrolled treated for median duration of 57.5 cycles (data cut-off 27 February 2021). Treatment with selumetinib showed confirmed response (cPR) (PR across two scans) in 75% and 68% of patients in phase I and phase II (stratum I), respectively. The response continued to be durable (lasting for >12 cycles) in approximately 60% of patients across both groups. Such significant and durable responses have not been seen historically with any medical therapy in PNs. Median time to initial response was 8 cycles and to best response was 18 cycles. Median PFS with a combined cohort was 88 cycles [85]. Similar results were seen in 25 patients enrolled on Phase II (stratum 2) with median 41 cycles. cPR was seen in 72% of patients with a durable response in 68%. Median time to best response was 24 cycles with the median PFS of this cohort not yet reached [86].

One of the biggest strengths of the SPRINT study lies in prospectively capturing patient-reported outcomes (PROs) and observed PROs in phase II (stratum 1 and 2) in the form of pain scale, the quality of life (QOL) and the global impression of change (GIC). Not only did patients show a sustained and durable response across the group on selumetinib but importantly showed a clinical benefit as demonstrated by improvement in PROs in almost 70% of patients after 1 year of treatment, which was maintained at 4 years. None of the patients on stratum II developed new PN-related symptoms while on selumetinib [86,88]. The impact of selumetinib, a MEK inhibitor, in transforming the treatment of inoperable plexiform neurofibromas (PNs) is evident in its swift clinical progression—advancing from a phase 1 trial in 2013 to FDA approval in 2020 in less than a decade.

Other MEKis currently under investigation for use in the treatment of NF1-PNs include binimetinib, trametinib and mirdametinib. Binimetinib is currently being evaluated in a phase II PNOC study of children with symptomatic PNs [89]. Preliminary data for 20 patients showed 75% partial response at 1-year with median maximal PN volume reduction of 25.5% [89], whereas preliminary data from phase I/IIa (NCT02124772) and phase II (NCT03363217) utilizing trametinib in pediatric patients with PNs showed ORR of 46% and 60%, respectively.

Mirdametinib is another oral MEK1/2 inhibitor that has had its activity demonstrated in PNs by a phase 2 study in participants older than 16 yrs where it showed PR in 42% of 19 patients at 12 cycles [83]. A recently published phase 2b ReNeu study further confirms this finding in a large cohort of 58 adults and 56 children, where 41% of adults and 52% of children achieved an objective response. The median-target PN volumetric best response was 41%, with children taking a median time of 13.4 month and adults taking 15.2 months to achieve the best response. Importantly, both cohorts reported a significant and clinically meaningful improvement in PRO (pain and health-related quality of life (HRQOL)) that had an early onset and sustained through the treatment. In total, 71% of adults and 79% of children at cycle 13 reported an improvement in the overall status [90].

### 5.2. Multiple Tyrosine Kinase Inhibitor

Cabozantinib is an oral tyrosine kinase inhibitor (TKI) that is approved for the treatment of adult patients with hepatocellular carcinoma or advanced renal cell carcinoma. Based on preclinical studies of cabozanitib where it reduced the number, volume and PN angiogenesis in Nf1 mutant mice, it was used in a phase II trial (NCT02101736) for patients more than 16 years old with PNs and demonstrated PR in 42% (8/19) [84].

Furthermore, it is important to note that NF1 patients with pLGGs have been excluded from some of the most recently published trials, such as dabrafenib and trametinib or current ongoing trials (LOGGIC/FIREFLY-2) for pLGGs, thus limiting treatment options for this cohort of patients. This may in turn lead to off-label use of these agents.

## 6. Toxicity

Targeted therapies have unique toxicity profiles that differ from those of conventional chemotherapy in pLGGs. Conventional chemotherapy is often associated with myelosuppression, alopecia, ototoxicity (carboplatin), neuropathy (vincristine), and reduced fertility potential (procarbazine and lomustine) [91,92]. Furthermore, some commonly used agents like lomustine, procarbazine and temodal are associated with the risk of myelodysplastic syndrome and secondary leukemia, which are often aggressive and difficult to treat [93].

### 6.1. MEK and BRAF Inhibitors

BRAFi and MEKi therapies share significant overlap in toxicity profiles. Skin toxicity is the most frequent AE, affecting up to 60%, and includes maculopapular rash, dry skin, photosensitivity, acne and alopecia [94]. Other reported AEs include elevated creatine phosphokinase (CPK), anemia, diarrhea, headache, nausea, fatigue, AST and ALT elevations, and hypoalbuminemia [7]. Less-frequent AEs include pyrexia (more common with BRAFi–MEKi combination), QT prolongation, hypertension, pericarditis, uveitis, arthralgias, fatigue, vomiting, diarrhea and/or mucositis [14,37].

Interestingly, skin-related toxicity can vary between the agents. In addition, combination therapy of dabrafenib/trametinib has demonstrated greater tolerability with fewer dose interruptions, reductions and discontinuations compared to MEKi monotherapy.

### 6.2. Pan-RAF Inhibitors

Treatment with Pan-RAF inhibitors shows overlapping toxicity to MEKis and BRAFis. Therapy with Pan-RAF inhibitors is associated with hair color changes (76%), elevated creatine phosphokinase (56%) and anemia (49%) being the most common treatment-related adverse events [18]. Within the FIREFLY-1 (PNOC026) trial, 42% of patients experienced grade 3 or greater toxicity, including elevated CPK (12%), anemia (10%) and rash (8%). Nine patients (7%) developed toxicity leading to discontinuation of tovorafenib. It is important to note that there has not been reported ocular toxicity, cardiac toxicity or weight gain with the use of tovorafenib, although tovorafenib has been associated with a decrease in growth velocity in some children, with no associated bone age advancement or premature growth plate fusion [18]. The recovery of growth velocity was noted after discontinuation of therapy, suggesting the potential for “catch-up” growth.

### 6.3. FGFR Inhibitors

FGFR inhibitors have been linked to specific toxicity, which often limits the use of these medications. The most common side effects include ocular toxicity (dry eyes, keratitis, diplopia and blurred vision), diarrhea, significant onycholysis and nail infections, palmar–plantar erythrodysesthesia syndrome, mouth sores, fatigue and elevated phosphate levels often requiring chelation [26,95,96]. More recently, a retrospective report including seven pediatric patients treated with an FGFR inhibitor raised concerns about slipped capital femoral epiphyses, which occurred in 42% of the patients [97].

### 6.4. TRK Inhibitors

Larotrectinib and entrectinib can be responsible for frequent toxicity, although most of the reported adverse events are grade 1 or 2 [98]. A large retrospective analysis using the US Food and Drug administration Adverse Event Reporting System (FAERS) database in a 4-year period (2019–2022) identified 807 individual case safety reports; of these, only 18 involved pediatric patients; 94% of these adverse events (AEs) in the pediatric populations were related to Larotrectinib. Of note, while pain and dizziness were the prominent side effects in adult patients, in keeping with the fact that TRK receptors are key regulators of pain and balance, pediatric AEs were heterogenous and not neurologic in nature. Instead in pediatric patients, AEs included gastrointestinal symptoms (nausea and vomiting), pyrexia, increased body weight, and blood count abnormalities [99]. When looking specifically at the pediatric CNS cohort, in a cohort of 33 patients, grade 3–4 toxicity was reported in 9% of patients, with no new side effect reported [27].

With regard to entrectinib, a large phase 1/2 trial has shown that the most frequent AEs were weight gain (occurring in almost 50% of the cohort), followed by anemia, creatinine elevation and gastrointestinal symptoms. Of note, 20% of the pediatric patients experienced the occurrence of bone fractures, an event that seemed to occur more frequently in pediatric patients [24,100].

### 6.5. mTOR Inhibitor

The most frequently reported toxicity of everolimus includes stomatitis, gastrointestinal symptoms (i.e., diarrhea), fatigue, leucopenia, anorexia and anemia. Of note, most of these AEs are grade 1 or 2 and are easily reversible [101]. Other frequent side effects include hyperlipidemia and hyperglycemia [102]. Rarer yet potentially lethal adverse events related to this drug are non-infectious pneumonitis and interstitial lung disease; thus, special attention has to be paid in the case of the occurrence of pulmonary symptoms during treatment [102,103].

## 7. Discussion

There have been significant advances in the treatment of glioma and NF-1 associated PNs over the past decade, which have been largely driven by the study of small molecule inhibitors. Defining the molecular drivers of gliomagenesis provided a gateway to introduce these agents into the therapeutic paradigm. These agents have demonstrated significant efficacy in MAPK-driven pLGGs and PNs, as highlighted by recent FDA approvals of dabrafenib/trametinib and selumetinib for the upfront treatment of inoperable pLGGs and NF1-associated PNs, respectively, in the pediatric population.

Despite this success, there are many outstanding questions. Firstly, the optimal duration of therapy is unknown. Most trials have used 18–24 months but allowed extended use for those with an ongoing benefit. In some cases, most notably for tumors harboring BRAF p.V600E, rapid progression can be seen in patients after stopping drug [17]. In other cases, progression may be more protracted but remains a risk. In the future, it is possible that monitoring strategies, using new diagnostic techniques such as liquid biopsy may be used to determine the appropriate duration of therapy. Secondly, targeted therapies have generally been used as a single agent. The current studies are ongoing to determine the safety and efficacy of combination therapy using targeted therapy combined with chemotherapy. Thirdly, while resistance is rare, the mechanism of resistance is unknown. Along this line, it has been shown that patients can re-respond to the same agent if progression occurs after the cessation of therapy, but whether the outcomes remain the same as the first exposure is unknown. Fourthly, given the excellent long-term survival, the goal of therapy in pLGGs is to reduce long-term morbidity. Many studies have attempted to collect functional outcomes, such as vision. Studies will be needed to look at long-term functional outcomes, remote from therapy, to determine if these are improved compared to historical cohorts to better understand durability of these outcomes. Lastly, given these medications are still relatively new within the treatment paradigms of glioma, ongoing surveillance for the identification of late effects in survivors will be needed.

As described, targeted therapy has changed treatment paradigms. This is evolving, with growing evidence to introduce targeted therapy at earlier time points in a patient’s treatment journey. While this improves outcomes compared to historical treatments such as chemotherapy, it comes at a significant financial cost, particularly considering the open-ended duration of therapy needed for some patients. Given the cost of these medications, it may be difficult to justify, even in some developed countries [104]. Unfortunately, this will lead to disparity in regard to global access to these medications, with those in lower-income countries with limited or no access to these therapies widening the gap in outcomes in childhood cancer between high and low-income countries.

Current studies have identified a clear role for targeted therapies in tumors harboring the common molecular alterations, but there are limited options for those with non-BRAF or NF-1-altered tumors, such as FGFR-altered glioma. Some studies have included these patients, while this has been an exclusion criterion for other studies. Further study is needed to better understand outcomes for these alterations.

The role of targeted therapy in the treatment of pHGGs is less well defined. While there are subgroups where targeted therapy has a clear role, such as pHGGs harboring BRAF p.V600E, treatment with a single agent is insufficient for disease control for other RAS/MAPK-altered pHGGs. Further work is needed to identify combination strategies to control these tumors.

## 8. Conclusions

Targeted therapy has provided new treatment options for patients with pLGGs and NF1-PNs, with the hope of reducing long-term morbidity due to their disease. These effective agents have changed the treatment paradigm for patients with improved outcomes compared to historical treatments such as chemotherapy. The same success has not been observed for the majority of RAS/MAPK-altered pHGGs, but targeted therapy does offer opportunities for combination therapy for future clinical trials.

## Figures and Tables

**Table 1:** Targeted therapy studies in pLGGs.

Targeted Therapy Study Information/Design	Target	Study Population	Response Rate/Outcome	Median Time to Response
Selumetinib [7,8] Phase I/II	MEKi	Non-NF1 progressive, recurrent, or refractory pilocytic astrocytoma with *KIAA1549::BRAF* or BRAF p.V600E (stratum 1)	32% CR or PR 40% SD 28% PD 2-year PFS 70%	Median time to PR 7.54 months
		NF1-associated progressive, recurrent, or refractory pLGGs (stratum 3)	36% CR or PR 60% SD 2-year PFS 96%.	3.57 months
		Non-NF1 recurrent, progressive, or refractory optic pathway and hypothalamic pLGGs (stratum 4)	24% PR 56% SD 2-year PFS 78%	
Trametinib [9] Phase I/II	MEKi	Relapsed/refractory BRAF p.V600E tumors	15% response rate PFS 16.4 months	
Trametinib and Dabrafenib [9] Phase I/II	MEKi and BRAFi	Relapsed/refractory BRAF p.V600E pLGG	Response rate 25% PFS 36.9 months	
Dabrafenib and Trametinib [10] Phase II	MEKi and BRAFi	Upfront/newly diagnosed BRAF p.V600E pLGG	ORR 47% PFS 20.1 months	
Trametinib [11] Phase II	MEKi	Progressive/refractory pLGGs with MAPK/ERK pathway activation. Patients with NF1 and without NF1	Ongoing study	
MEK162 [12] (binimetinib) Phase II	MEKi	Recurrent or progressive pLGGs with *BRAF* fusion, NF1-associated pLGGs and those without documented *BRAF* fusion	56% radiographic response	
Cobimetinib [13] Phase I/II	MEKi	Pediatric and young adult patients with relapsed or refractory solid tumors	ORR 5.4% Median PFS 20.2 months	
Selumetinib Randomized, prospective Children’s Oncology group (COG) phase III study (NCT04166409/NCT03871257 or ACNS1831/1833)	MEKi	Newly diagnosed and previously untreated optic pathway/hypothalamic gliomas with and without NF1		
Dabrafenib Phase I/II [14]	BRAFi	Recurrent/refractory pLGGs with BRAF p.V600E	ORR 44% 1-year PFS 85%	3.8 months
Vemurafenib [15] Phase I	BRAFi	Recurrent/refractory BRAF p.V600E pLGGs	1 patient CR, and 5 PR, with the remaining 13 SD	
Vemurafenib [16] Retrospective	BRAFi	BRAF p.V600E LGGs (upfront or relapse/recurrence)	57% response rate	Time to best radiographic response 3 months,
Dabrafenib or Vemurafenib [17] Retrospective	BRAFi	BRAF p.V600E gliomas (upfront or relapse/recurrence)	Objective radiographic responses 80% pLGGs. PFS at 2 years was 81.6%	Average time to response 4 months.
Tovorafenib [18] Phase II	Next-generation RAFi	*BRAF*-altered relapsed or refractory pLGG	ORR 67% by RANO-HGG criteria ORR 51% by RAPNO	5.3 months
Tovorafenib monotherapy [19] LOGIC/FIREFLY-2 trial Phase III—randomized trial of tovorafenib vs. chemotherapy (VCR/carbo or vinblastine)	Next-generation RAFi	Upfront pLGG		Ongoing study
Vinblastine with Tovorafenib VICTORY (NCT06381570)	Next-generation RAFi	Recurrent/progressive RAF-altered pLGG		Ongoing study
Plixorafenib [20] Phase 1/2	Next-generation RAFi	Recurrent/progressive BRAF-altered solid tumors	42% ORR	
Plixorafenib Phase II NCT05503797	Next-generation RAFi	Children or adults with BRAF-altered tumors		Ongoing study
Sorafanib [21] Phase II		Relapsed or refractory pediatric low-grade astrocytoma	Radiographic progression in 9 (82%) patients, requiring discontinuation of sorafenib	Not reported
Everolimus [22] Phase II	mTOR	Progressive pLGG	Response rate 52.2% 2, 3 and 5-year PFS were 39 ± 11%, 26 ± 11% and 26 ± 11%, respectively, 2, 3 and 5-year OS 93 ± 6%	0.9 months
Everolimus (Phase 1-2 trial)	mTOR	Children 3 years older with progressing subependymal giant-cell astrocytomas (SEGA)	Tumor volume reduced by at least 30% in 21 patients (75%); reductions of 50% or more in 9 patients (32%)	At the 3-month mark, tumor reduction in 27/28 patients
Trametinib and everolimus PNOC phase I trial (NCT04485559)	MEKI/mTOR	Recurrent LGGs and HGG		Ongoing study
Hydroxychloroquine with trametinib and/or dabrafenib Phase I/II trial PBTC-055 (NCT04201457)	MEKi/BRAFi	Recurrent pLGGs or pHGGs with BRAF alterations		Ongoing study
Larotrectinib [23] (retrospective)	TRK	Patients with TRK fusion-positive solid and CNS tumors	ORR of 68.8% in the pediatric population	Median overall survival of 185.5 months in the pediatric population
STARTRK-NG [24] (NCT 02650401) phase 1-2 trial	ALK, ROS-1, NTRK1-3	Pediatric or CNS tumors with either an ALK, a ROS1, or a NTRK 1-3 rearrangement	ORR 57.7%	Not available
Erdafitinib [25] (phase 2 COG Match trial)	FGFR 1-4	Arm B (APEC1621B) evaluated the efficacy of erdafitinib in patients aged 1–21 whose tumors harbored a FGFR 1-4 rearrangement	Partial response or stable disease in 54% of the patients with an FGFR 1 fusion	6-month OS of 89.7%
Erdafinitib [26] (RAGNAR phase 2 trial)	FGFR1-4	Patients 12 years of older whose solid or CNS tumor harbored a FGFR1-4 mutation	No response in the CNS low-grade tumor subgroup	-
Larotrectinib [27] (combined analyses of three phase 1/2 clinical trials)	TRK	Pediatric and adult patients with TRK fusion-positive solid and CNS tumors	ORR of 30%	At the 1-year mark, 55% of the patients remained progression-free

Complete response (CR) or partial response (PR), stable disease (SD), progressive disease (PD), minor response (MR), overall response rate (ORR), progression-free survival (PFS) and overall survival (OS) subependymal giant-cell astrocytoma (SEGA).

**Table 2:** Targeted therapy studies in pHGGs.

Targeted Therapy Study Information/Design	Target	Study Population	Response Rate/Outcome	Median Time to Response
Selumetinib [58] Pediatric MATCH		Relapsed or refractory solid tumors, lymphomas and histiocytic disorders	No objective response 2 pHGGs showed SD 6-month PFS 15%	
Dabrafenib or Vemurafenib [17] Retrospective	BRAFi	BRAF p.V600E-mutated gliomas (upfront or relapse/recurrence)	36% of pHGGs responded to BRAF inhibition, with all except one progressing within 18 months PFS at 2 years 81.6%	4 months
Dabrafenib [59] Phase I/II	BRAFi	Refractory, recurrent or progressive BRAF p.V600E pHGG	ORR was 45%	
Dabrafenib and Trametinib [60] Phase II	BRAFi/MEKi	Relapsed/refractory BRAF p.V600E pHGG	ORR 56% by RANO criteria Median PFS 9 months, OS 32.8 months	4 months
BRAF +/− MEK inhibitors [61] Retrospective	*BRAFi/MEKi*	*BRAF*-mutated pHGGs (upfront therapy)	13 patients with measurable disease and imaging available for central review, 8 responses (2 with CR, 6 with PR) 3-year PFS 65% and OS 82%	Median time to best response of 2.5 months
Dabrafenib and Trametinib (NCT03919071)	BRAFi/MEKi	Newly diagnosed BRAF p.V600E pHGG		Ongoing study
Trametinib and everolimus. PNOC phase I trial (NCT04485559)	MEKi	Recurrent pLGGs and pHGG		Ongoing study
Hydroxychloroquine with trametinib and/or dabrafenib. Phase I/II trial PBTC-055 (NCT04201457)	MEKi	Recurrent pLGGs or pHGGs with BRAF alterations		Ongoing study
Larotrectinib [23] (retrospective)	TRK	Patients with TRK fusion-positive solid and CNS tumors	ORR of 68.8% in the pediatric population	Median overall survival of 185.5 months in the pediatric population
Larotrectinib [27] (combined analyses of three phase 1/2 clinical trials)	TRK	Pediatric and adult patients with TRK fusion-positive solid and CNS tumors	ORR of 30%	At the 1-year mark, 55% of the patients remained progression-free
STARTRK-NG (NCT 02650401) phase 1-2 trial	ALK, ROS-1, NTRK1-3	Pediatric or CNS tumors with either an ALK, a ROS1 or a NTRK 1-3 rearrangement	ORR 57.7%	Not available
Repotrectinib (phase 1-2 open label study)	ALK, ROS-1, NTRK1-3	Pediatric and adult patients with advanced or metastatic malignancies harboring ALK, ROS-1 or NTRK 1-3 mutations	-	Ongoing study
BIOMEDE 2.0 (Randomized, open-label, muticenter study (ONC 201 vs. everolimus)	K27M mTOR	DIPG/DMG K27-mutated	-	Ongoing study

Complete response (CR) or partial response (PR), stable disease (SD), progressive disease (PD), minor response (MR), overall response rate/objective response rate (ORR), progression-free survival (PFS), and overall survival (OS).

**Table 3:** Targeted therapy studies in NF1-PNs.

Study (N)		Target	Study Population	Response Rate/Outcome	Median Time to Response
Selumetinib NCT01362803	Phase I [28]	MEK	Inoperable symptomatic PN	cPR: 75% PFS: 52 cycles	Best: 22 cycles
	Phase II [58] stratum 1		Age: 2–18 yrs Inoperable symptomatic PN	cPR: 68% PFS: not reached	Best: 16 cycles
	Phase II [8] stratum 2		Age: 2–18 yrs Inoperable asymptomatic PN	cPR: 72% PFS: not reached	Best: 24 cycles
Binimetinib [12]			Age: 2–16 yr pediatric cohort	PR: 74%	Not reported
Mirdametinib	NCT02096471 [83]		Age: >16 yrs Inoperable progressive/morbid PN	PR: 42%	
	ReNeu [36] (58 adults, 56 children)		Children: 2–17 yrs, adults >18 Inoperable PNs with morbidity	Children PR: 52% Adult: PR: 41%	Children: 13.4 m Adult: 15.2 m
Trametinib	(NCT02124772) 26 PN		Age: 1 m–18 yrs Inoperable medically significant PN	PR: 46%	Not reported
	TRAM-01 [11] (30 PN) NCT03363217		Age: 1 m–25 yrs Inoperable progressive/symptomatic PN	PR: 62.5%	Not reported
Cabozantinib [84] NCT02101736		TKI	Age: >16 yrs Inoperable progressive/symptomatic PN	PR: 42%	Not reported

cPR: confirmed partial response (>20% volume reduction in 2 scans > 3 months apart), PR partial response, PFS: progression-free survival and 1 cycle: 4 weeks.
